# Activation of WNT7b autocrine eases metastasis of colorectal cancer via epithelial to mesenchymal transition and predicts poor prognosis

**DOI:** 10.1186/s12885-021-07898-2

**Published:** 2021-02-19

**Authors:** Shuai Jiang, Qiwen Li, Yimin Liu, Huimin Zhang, Qianyu Wang, Yu Chen, Xiaoyang Shi, Jun Li, Hailing Zhang, Yi Zhang, Dongqing Xia, Man Wu, Jiajia Lin, Chenglin Zhang, Suhua Pang, Jiamin Jiang, Yan Wen, Peipei Zhang

**Affiliations:** 1 Geriatrics Department, Suqian First Hospital, No. 120, Suzhi Road, Sucheng District, Suqian, Jiangsu Province 223899 PR China; 2 Prenatal Diagnosis Center, Suqian First Hospital, No. 120, Suzhi Road, Sucheng District, Suqian, Jiangsu Province 223899 PR China; 3 Oncology Department, Suqian First Hospital, No. 120, Suzhi Road, Sucheng District, Suqian, Jiangsu Province 223899 PR China; 4 Department of Pathology, Suqian First Hospital, No. 120, Suzhi Road, Sucheng District, Suqian, Jiangsu Province 223899 PR China; 5 Department of General Surgery, Suqian First Hospital, No. 120, Suzhi Road, Sucheng District, Suqian, Jiangsu Province 223899 PR China; 6 Neurology Department, Suqian First Hospital, No. 120, Suzhi Road, Sucheng District, Suqian, Jiangsu Province 223899 PR China; 7 Department of Gastroenterology, Suqian First Hospital, No. 120, Suzhi Road, Sucheng District, Suqian, Jiangsu Province 223899 PR China

**Keywords:** WNT7b, Wnt/β-catenin signaling pathway, Colorectal cancer (CRC), Metastasis

## Abstract

**Background:**

Aberrant activation of the Wnt/β-catenin signaling pathway is one of the most frequent abnormalities in human cancer, including colorectal cancer (CRC). Previous studies revealed pivotal functions of WNT family members in colorectal cancer, as well as their prognostic values. Nevertheless, the prognostic role and mechanisms underlying WNT7b in colorectal cancer development remains unclear.

**Methods:**

In this study, WNT7b expression was measured by immunohistochemical staining of 100 cases of surgically resected human colorectal cancerous tissues as well as matched adjacent normal tissues constructed as tissue microarrays. In vitro studies, we attempted to substantiate the WNT7b expressional pattern previously found in immunohistochemistry staining. We used the colorectal cancer cell-line HCT116 and normal colorectal cell-line FHC for immunofluorescence staining and nuclear/cytoplasmic separated western blotting. We measured epithelial–mesenchymal transition (EMT) markers and migration capacity of HCT116 in the context of WNT7b knocked-down using short interfering RNA. Finally, clinical and prognostic values of WNT7b activation levels were examined.

**Results:**

WNT7b was expressed in the nucleus in adjacent normal tissues. In CRC tissues, nuclear expression of WNT7b was similar; however, membrane and cytoplasmic expression was strikingly enhanced. Consistently, in vitro analysis confirmed the same expression pattern of WNT7b. Compared with FHC cells, HCT116 cells displayed higher levels of WNT7b membrane and cytoplasmic enrichment, as well as higher migration capacity with a sensitized EMT process. Either partial knockdown of WNT7b or blockade of the Wnt/β-catenin signaling pathway reversed EMT process and inhibited the migration of HCT116 cells. Finally, elevated secretion levels of WNT7b were significantly associated with lymphatic and remote metastasis and predicted worse prognosis in the CRC cohort.

**Conclusion:**

In summary, we demonstrated that the activation of WNT7b autocrine probably contributes to CRC metastasis by triggering EMT process through the Wnt/β-catenin signaling pathway. High levels of WNT7b autocrine secretion predicts poor outcome in patients with CRC. This molecule is a promising candidate for clinical CRC treatments.

**Supplementary Information:**

The online version contains supplementary material available at 10.1186/s12885-021-07898-2.

## Background

Colorectal cancer (CRC) is one of most prevalent malignant neoplasms of the digestive system [1]. Therapeutic approaches to CRC include surgical removal of polyps and early diagnosis, and these have been reported to improve prognosis and prolong survival [2]. Nevertheless, the treatment and prevention of CRC remains a global challenge because of the high risk of invasion and metastasis during CRC progression [3]. Recurrences of cancer in the liver, lung or peritoneal are major outcomes of CRC treatment failure. Therefore, the identification of critical molecules regulating CRC metastasis is urgently needed to improve treatment.

Epithelial–mesenchymal transition (EMT) is a developmental process by which epithelial cells are converted to mesenchymal cells during embryogenesis, tissue remodeling, wound healing, and tumor metastasis [4, 5]. During EMT, epithelial cells acquire mesenchymal cell properties and show reduced intercellular adhesion and increased invasion [5]. In cancer cells, EMT is abnormally regulated by extracellular stimuli derived from the tumor microenvironment, including growth factors and inflammatory cytokines, along with intra-tumoral physical stresses such as hypoxia [6]. EMT programming allows tumor cells to adapt to the constant changes of the tumor microenvironment, in so doing to successfully metastasize.

The wingless/integrase-1 (Wnt) pathway is a major homeostatic signaling cascade in development and stem cell homeostasis [7, 8]. In the canonical or β-catenin–dependent signaling branch, secreted Wnt ligands engage a transmembrane receptor system consisting of the Frizzled family core and LRP5/6 co-receptors to inhibit a multiprotein β-catenin destruction complex [9]. Consequently, cytosolic β-catenin is relieved from constitutive proteasomal degradation and induces the transcription of target genes through association with TCF/LEF family transcription factors. Specificity of the Wnt signaling output is achieved primarily via the differential expression of a wide range of Wnt ligands and receptors that exert overlapping but nonredundant functions [10].

The expression of WNT7B and its co-receptors are largely restricted to specific tissues, especially the developing brain, where they contribute to blood–brain barrier formation and maintenance through activation of Wnt/β-catenin signaling [11, 12]. Increased expression of WNT7B and subsequent Wnt pathway activation have been observed in several cancers, including prostate cancer [13], pancreatic cancer [14], and breast cancer [15]. Nevertheless, as is the case with most Wnt ligands, it remains largely unresolved how WNT7b expression is regulated as well as its underlying mechanisms in CRC.

Therefore, in the present study, we measured WNT7b expression in CRC using immunohistochemistry staining (IHC) and described the expression patterns of WNT7b, and further verified these using in vitro studies.

## Methods

### Ethics statement

This study was reviewed and approved by Medical Ethics Committee of the Suqian First People’s Hospital.

### Patients and human tissue samples

Human CRC tissue microarrays (TMAs) were constructed from 100 cases of surgically resected colorectal tumor tissues (2016–2018), along with matched cases of adjacent normal tissues from the Pathology Department, Suqian First Hospital, Jiangsu Province, China. All tissue specimens were reviewed using hematoxylin and eosin staining; representative areas free from necrosis and hemorrhage were selected in the paraffin blocks. We took 1-mm diameter cylinders from intratumoral or peritumoral tissues (1–2 cm from the tumor edge) and transferred to the TMA by the Outdo Biotech Company, Shanghai, China. The relevant clinical data was collected using retrospective medical chart reviews. Survival data were collected every 3 months, with the final update on 10/31/2019. All protocols were reviewed and approved by the academic ethics committee.

The demographic data and post-surgical follow-up of the 100 CRC cases are shown in Table 1. The majority of patients were diagnosed (post-surgically) with stages II and III according to the American Joint Committee on Cancer (AJCC) staging 8th edition (72/100), 19 cases were diagnosed as AJCC stage I, and nine cases were AJCC stage IV as have been found with liver metastasis prior to the surgical resection.

**Table 1:** CRC cohort parameters

Patient characteristics (***n*** = 100)		
Characteristic	Sub-characteristic	Value
Age		66 (range 43–89)
Gender	Male	59
	Female	41
Depth of invasion (T)	T2	35
	T3	63
	T4	2
Lymph node metastasis (N)	N0	39
	N1	55
	N2	6
Distant metastasis (M)	M0	91
	M1	9
AJCC stage	I	19
	II	38
	III	34
	IV	9
Total		100

### Immunohistochemistry and scoring

Archived paraffin-embedded tumor tissues and adjacent normal tissues were constructed for tissue microarrays and IHC. IHC was performed using the polymer HRP detection system (Zhongshan Goldenbridge Biotechnology, Beijing, China) according to the manufacturer’s instructions. The primary antibody was anti-WNT7b (Catalog#: AF3460, R&D system) at 1:1000 dilution. The second antibody, was anti-goat (Catalog#: 6403–05, BioVision) at 1:2000 dilution. All TMA slides were scanned by the resolution of 40x: 0.25 μM/pixel using the Leica Aperio AT2 digital slide scanner (Leica Biosystems, IL, US).

The scoring of WNT7b immunostaining was based on both the intensity and percentage of positively membrane/cytoplasmic staining cells. The final expression score of a single sample was equal to the intensity grade (0, 1, 2, and 3) multiplied by the percentage level (0–100%). Each CRC sample was compared with its paired adjacent normal tissue. TMA slides was evaluated by two independent pathologists who were blinded to patient information. If there were discrepancies, results were jointly assessed by both investigators and the final score was formed by consensus.

### Cell cultures

HCT116 cells were purchased from Tongpai Biotechnology Company, Shanghai, China. FHC cells were purchased from Suran Biotechnology Company, Shanghai, China. Cells were maintained in 5% CO_2_ at 37 °C in Dulbecco’s Modified Eagle’s Medium (DMEM, Life Technologies/Gibco, NY, USA) supplemented with 10% fetal bovine serum (FBS, Life Technologies/Gibco, NY, USA), 100 U/mL penicillin, and 100 mg/mL streptomycin (Life Technologies/Gibco, MD, USA). Cells (1 X 10^6^) were seeded into dishes (10-cm diameter) for 72 h at each passage.

### Transient transfection

For transient *WNT7b* gene knockdown, cell-lines were transfected with 10–20 nM of duplexed siRNAs using Lipofectamine 2000 (Invitrogen/Life Technologies, NY, USA). The RNA interference sequence is listed in the Supplementary Data 2. The specificity of the knock-down of WNT7b was validated by qRT-PCR analysis (data was shown in Supplementary Data 2).

### Western blot

Cells were harvested at 90% confluence. For regular immunoblots, cell lysates were obtained using RIPA Lysis Buffer (Millipore, MA, USA). Separation of the nuclear, cytoplasmic, and membrane protein was performed by Membrane, Nuclear and Cytoplasmic Protein Extraction Kit (BIO BASIC, Markham ON, Canada) following the indicated instructions. Cell lysates were separated by 10% sodium dodecyl sulfate–polyacrylamide gel electrophoresis and transferred to polyvinylidene fluoride membranes (Millipore, MA, USA). The membranes were probed with primary antibodies overnight at 4 °C. Membranes were incubated with horseradish peroxidase-conjugated secondary antibodies for 1 h at room temperature. The immune complexes were detected using enhanced chemiluminescence (Cell Signaling Technology, MA, USA). GAPDH was used to correct for differences in loading of the proteins from the control and experimental groups. All experiments were repeated for three times independent analysis and quantified by image J. Detailed antibody information is displayed in Supplementary Data 1 and full-length blots/gels are presented in Supplementary Figure 1.

### Immunofluorescence assay

Cells grown on cover slips in 24-well plates were fixed in 4% paraformaldehyde for 20 min, then the cells were permeabilized with 0.5% Triton X-100 (Solarbio, Beijing, China) for 20 min at room temperature. The HCT116 cells and FHC cells were incubated at 4 °C overnight with the primary antibody (Catalog #: AF3460, 1:1000 dilution, R&D system) after blocking with 3% nonfat dry milk in PBS for 1 h at room temperature. Then the cells were incubated for 1 h at 37 °C with Fluorescein (FITC)-conjugated AffiniPure Fab Fragment (1:200 dilution, Jackson ImmunoResearch Laboratories, PA, USA) as the secondary antibody. The nuclei were stained by DAPI (Sangon Biotech, Shanghai, China) for 5 min after washing with PBS. Images were captured using a confocal microscope (Olympus, Tokyo, Japan).

### Trans-well assay

Trans-well assays were performed using growth factor-reduced, Matrigel-coated filters (8 mm pore size, BD, Franklin Lakes, NJ, US) in 24-well plates. Cells were trypsinized and seeded onto the upper chambers of the Trans-wells (3 × 10^4^ cells/well) in supplement-free DMEM medium. The lower chambers of the Trans-wells were filled with DMEM medium containing 100 ng/mL of EGF. The chambers were incubated at 37 °C with 5% CO_2_ for 24 h. At the end of incubation, cells on the upper surface of the filter were removed using a cotton swab. Cells migrating through the filter to the lower surface were fixed with 4% paraformaldehyde for 10 min and stained with 0.1% crystal violet for 5 min. Migrated cells were viewed and photographed using a phase-contrast microscope (Olympus).

### Statistical analyses

Two tails student’s t test was used for analysis of all the Western Blots. The χ^2^ test was used to analyze the significance of migrated cells in the Transwell assay using SPSS. Fisher’s exact test were used to analyze the significance of lymphatic and remote metastasis between each group based on membrane/cytoplasmic upregulated expression of WNT7b by SPSS. For survival analysis, we compared Kaplan–Meier survival curves between different thresholds of membrane/cytoplasmic upregulated expression of WNT7b using the log-rank test on GraphPad Prism 7.

## Results

### IHC labeling of WNT7b expression pattern in CRC and adjacent normal tissues

**Fig. 1:**
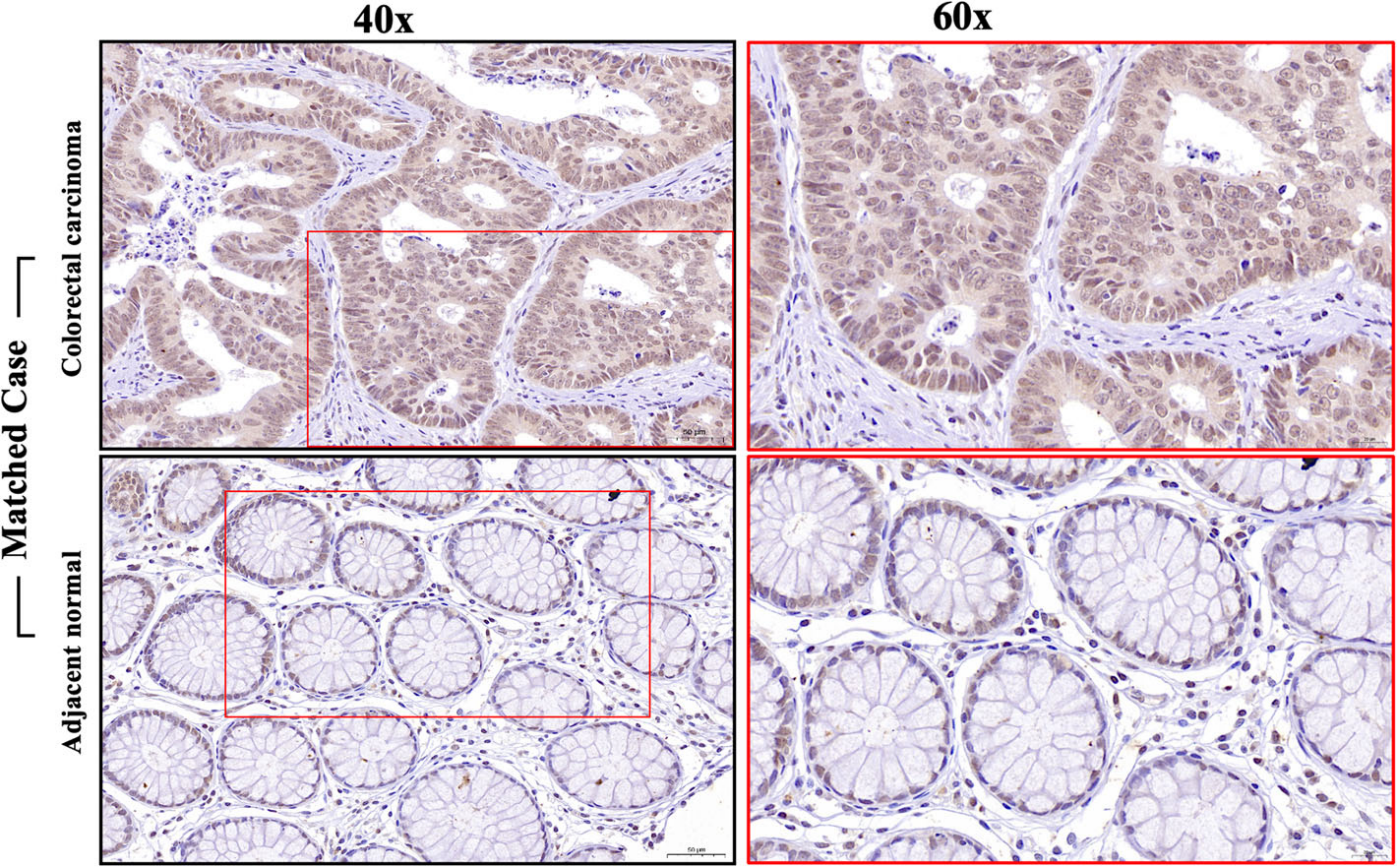
Expressional pattern of WNT7b in colorectal adenocarcinoma and matched adjacent normal specimen. WNT7b IHC binding detail in colorectal carcinoma tissue and matched adjacent normal tissue. Scale bar, 40x: 50 μm; 60x: 20 μm

**Fig. 2:**
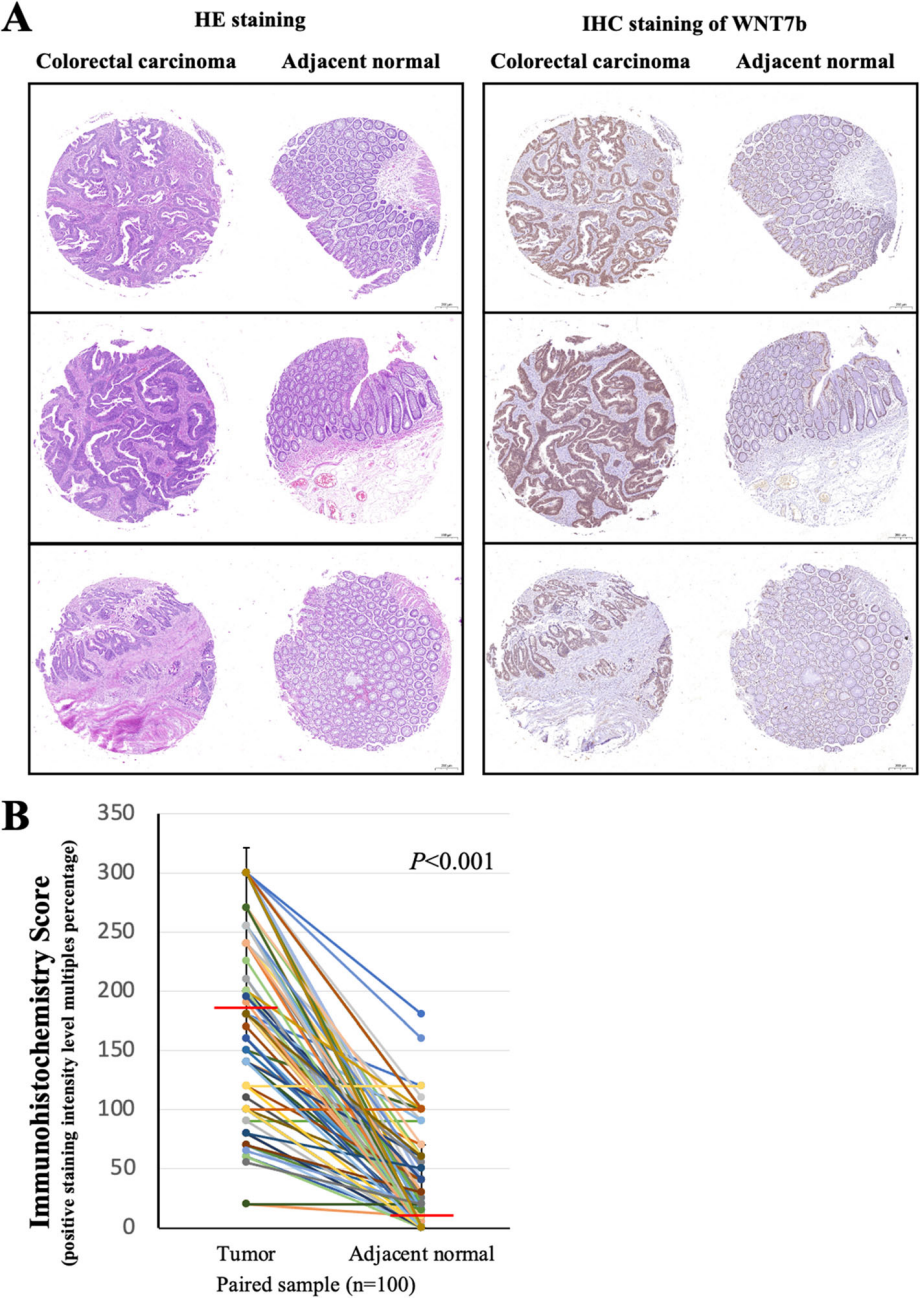
WNT7b expression was upregulated in CRC tissue. **a** Representative specimens of TMA slides showing H&E staining and WNT7b IHC binding pattern. Scale bar, 20 μm. **b** Quantification of the membrane/cytoplasm WNT7b expression in paired samples (tumor tissue and adjacent normal tissue, *n* = 100). Red line shows median level of IHC scoring. *P* < 0.001, student’s t test

To evaluate the expression of WNT7b in primary CRC, we performed immunohistochemical staining of TMA slides of both CRC and adjacent normal tissues (Catalog #: AF3460, R&D system, 1:1000 dilution) (Fig. 1). In adjacent normal tissues, WNT7b was expressed in the nucleus (99/100, 99%). Weak and sporadic positive membrane/cytoplasmic staining was also observed in 67 peritumoral cases (67/100, 67%). However, in CRC tissues, nuclear expression levels of WNT7b were similar; interestingly, membrane/cytoplasmic expression levels were strikingly enhanced. All samples presented positive membrane/cytoplasmic WNT7b staining (100/100, 100%). Partial cases of the matched IHC staining of WNT7b as well as the H&E staining were shown in Fig. 2a and the quantification of the membrane/cytoplasmic staining was shown in Fig. 2b, WNT7b expression was significantly upregulated compared with the matched adjacent normal tissue (*P* < 0.001). These result suggest that WNT7b autocrine secretion may have been activated during colorectal cell transformation.

### CRC cells showed enhanced WNT7b autocrine secretion

**Fig. 3:**
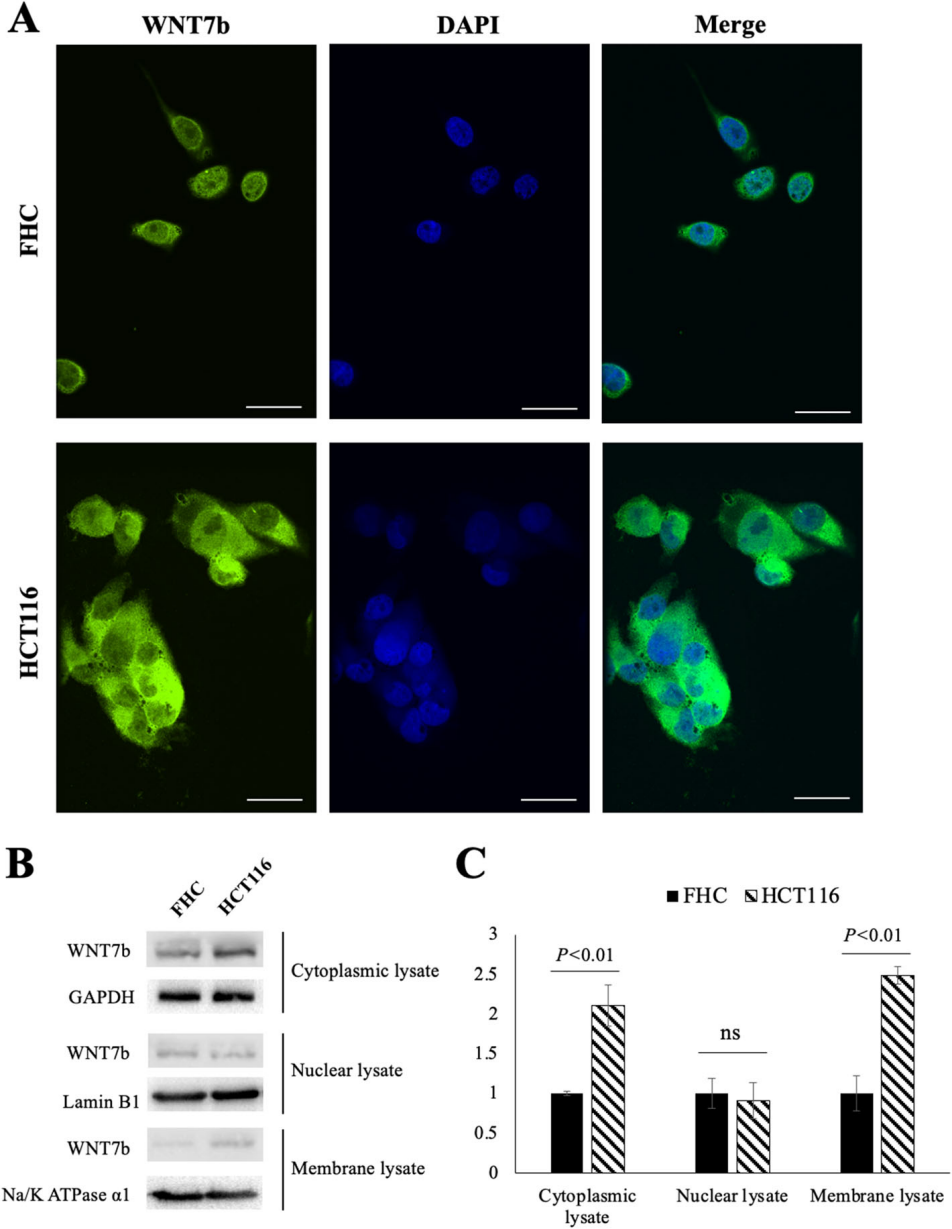
WNT7b was overexpressed in colorectal cells. **a** Representative images of immunofluorescence staining of WNT7b subcellular localization of FHC and HCT116 cells. Nuclei were counterstained with DAPI. Scale bar equals to 20 μm. **b** and **c** Cytoplasmic, nuclear, and membrane lysate of FHC and HCT116 cells were separately extracted and analyzed by Western Blot, with GAPDH, Lamin B1, and Na/K ATPase alpha 1 as the loading control, respectively. Quantification of the WNT7b expression was analyzed by image J, *P* value was shown and calculated by student’s t test

To confirm the WNT7b expression pattern found on IHC staining, the colorectal cancer cell-line HCT116 and the normal colorectal cell-line FHC were selected for subsequent studies. First, using immunofluorescence staining, we observed that membrane/cytoplasmic WNT7b expression was dramatically higher in the CRC cell-line HCT116 than in the normal colorectal cell-line FHC (Fig. 3a). Western blotting analysis confirmed this feature when we split the nuclear, cytoplasmic and membrane protein lysates (Fig. 3b and c). In the nuclear lysate, similar levels of WNT7b expression were detected between HCT116 and FHC cells while cytoplasmic and membrane WNT7b expression levels were elevated in HCT116 cells. These findings confirmed the WNT7b expression pattern in CRC and also suggested that high level of WNT7b autocrine secretion may stimulate malignant transformation of colorectal cells.

### WNT7b promotes cell migration by inducing EMT in HCT116 cells

**Fig. 4:**
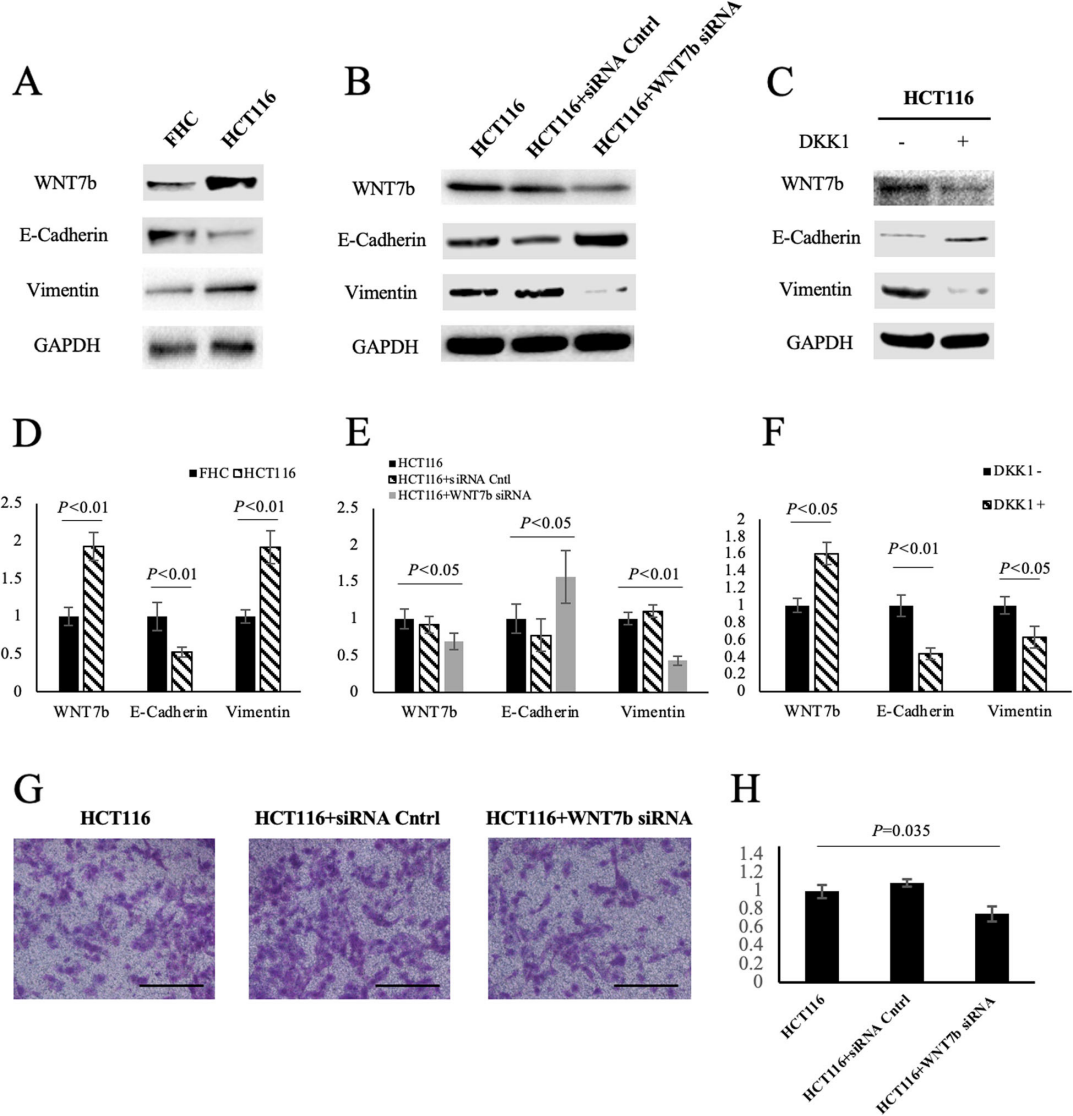
WNT7b was associated with epithelial to mesenchymal transition in CRC cells. **a** and **d** Western blot analysis detecting epithelial to mesenchymal transition marker E-Cadherin and Vimentin protein expressed in normal colorectal cells (FHC) and colorectal cancer cells (HCT116). **b** and **e** WNT7b was knocked down by transiently transfection of short interfering RNA of HCT116 cells. **c** and **f** The Wnt signaling pathway was inhibited by its antagonist Dickkopf-1 (DKK1). **g** Trans-well assay analyses of the migratory capacity changes as WNT7b was partially knocked down in HCT116 cells. Bars, 500 μm. **h** Quantification of migrated cells of each group based on three randomly chosen fields, χ^2^ test

It is unclear as to whether WNT7b is involved in canonical β-catenin-dependent signaling, or the self-dependent signaling pathway in CRC. This is because WNT7b and its co-receptors depending on the tissue. We initially checked how WNT7b was involved in canonical Wnt/β-catenin pathway in CRC cells. Because the canonical Wnt/β-catenin pathway is known to drive EMT, we firstly compared EMT markers between FHC cells and HCT116 cells. As shown in Fig. 4a and d, HCT116 cells presented higher WNT7b expression levels with activated EMT compared with FHC cells. Moreover, partial knockdown of WNT7b expression using small interfering RNA reversed the EMT process in HCT116 cells (Fig. 4b and e). Additionally, Wnt/β-catenin pathway-specific antagonist Dickkopf-1 (DKK1) was used to determine whether this reversion was regulated by the Wnt/β-catenin pathway. By blocking the Wnt/β-catenin pathway, HCT116 cells exhibited decreased WNT7b expression and inhibited EMT (Fig. 4c and f). These findings suggest that WNT7b is associated with EMT in CRC cells via the Wnt/β-catenin pathway. Finally, knock-down of WNT7b significantly downregulated the migratory capacity of HCT116 cells (Fig. 4g and h). These findings suggest that WNT7b is involved in promoting cells migration via the Wnt/β-catenin pathway and by enhancing EMT.

### Upregulated WNT7b was significantly associated with lymphatic and remote metastasis in the CRC cohort

**Fig. 5:**
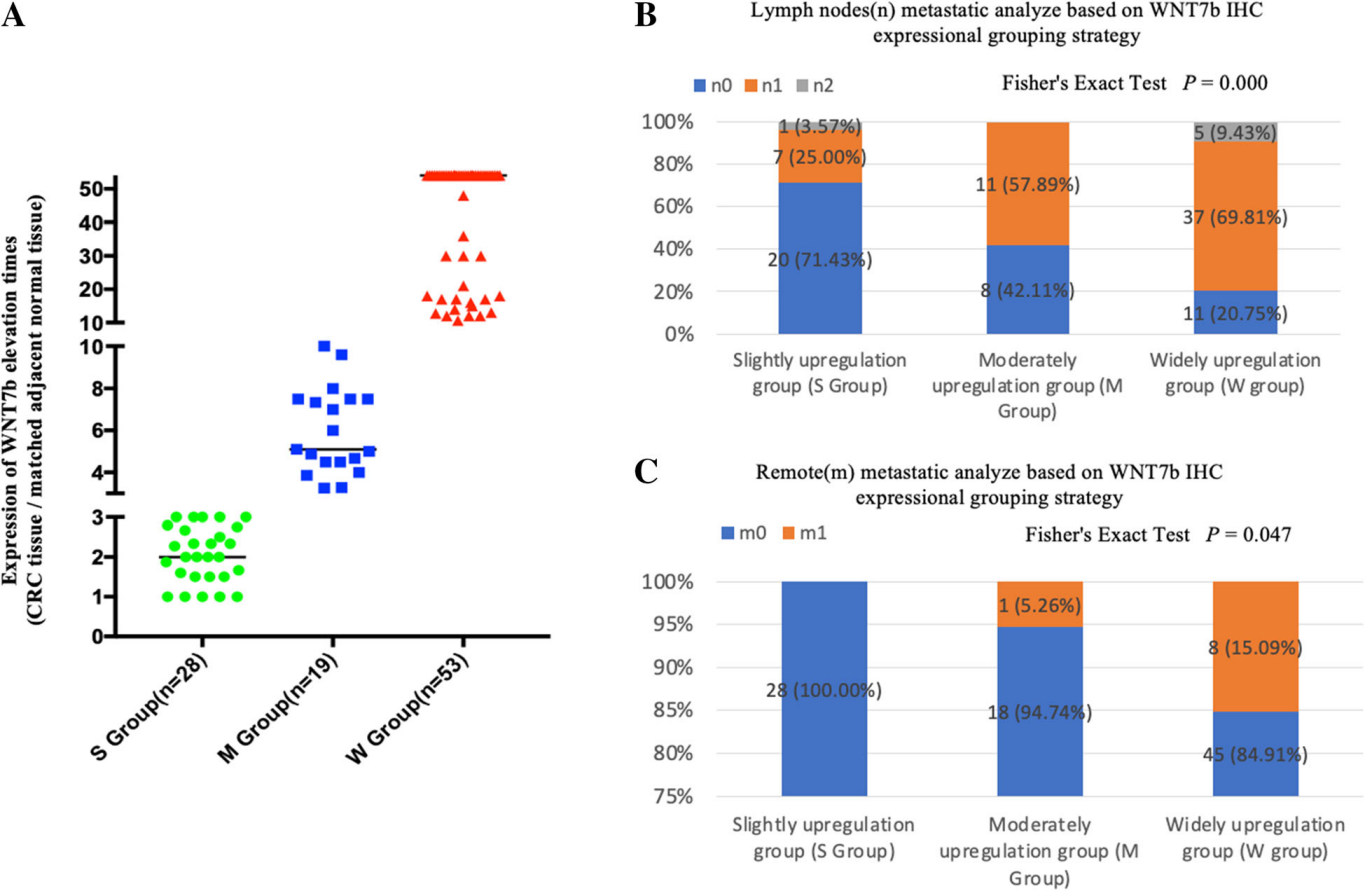
Membrane/cytoplasmic enrichment of WNT7b was significantly associated with Lymphatic and remote metastasis in CRC cohort. **a** CRC Cohort was divided into three subgroups based on multiple WNT7b elevation levels (compared with matched adjacent normal tissue) as Slightly upregulation group (S group), Moderately upregulation group (M group), and Widely upregulation group (W group) (See WNT7b IHC scoring and grouping strategy detail in Result section). **b** and **c** Statistical analysis of upregulated WNT7b expression was significantly associated with lymphatic and remote metastasis rate. *P* was shown and calculated by Fisher’s Exact Test

Because the function of WNT7b in manipulating CRC cell migration was identified, we further studied the correlation between WNT7b membrane/cytoplasmic enrichment level and metastatic rates in CRC patients. The CRC cohort was divided into three subgroups based on following strategy: Because each sample was assessed by comparing the WNT7b labeling score in CRC tissue with paired adjacent normal tissue, the grouping strategy was based on varied WNT7b upregulation scales. The classifications were the Slight Upregulation Group (1 ≤ elevated times ≤3, S Group, *n* = 28), the Moderate Upregulation Group (3<elevated times ≤10, M Group, *n* = 19), and the Widely Upregulation Group (10<elevated times, W Group, *n* = 53) (Fig. 5a). We performed correlation analyses between WNT7b upregulation and the TNM stages of CRC patients (Table 2). Statistical analysis suggested that high levels of WNT7b upregulation were significantly related to lymphatic (*p* = 0.000) and remote (*p* = 0.047) metastasis (Fig. 5b and c). This finding was consistent with in vitro studies that showed that abnormally upregulated WNT7b autocrine secretion was associated with CRC cell migration, thereby enabling metastasis of CRC.

**Table 2:** Correlations between WNT7b upregulation and TNM stages of CRC patients

	S Group	M Group	W group	
T2	11	1	23	
T3	16	17	30	P(T) = 0.133
T4	1	1	0	
N0	20	8	11	
N1	7	11	37	P(N) = 0.000
N2	1	0	5	
M0	28	18	45	P(M) = 0.047
M1	0	1	8	

*P* value was calculated by Fisher’s Exact Test

### WNT7b expression was related with poor post-surgical survival rates in CRC patients

**Fig. 6:**
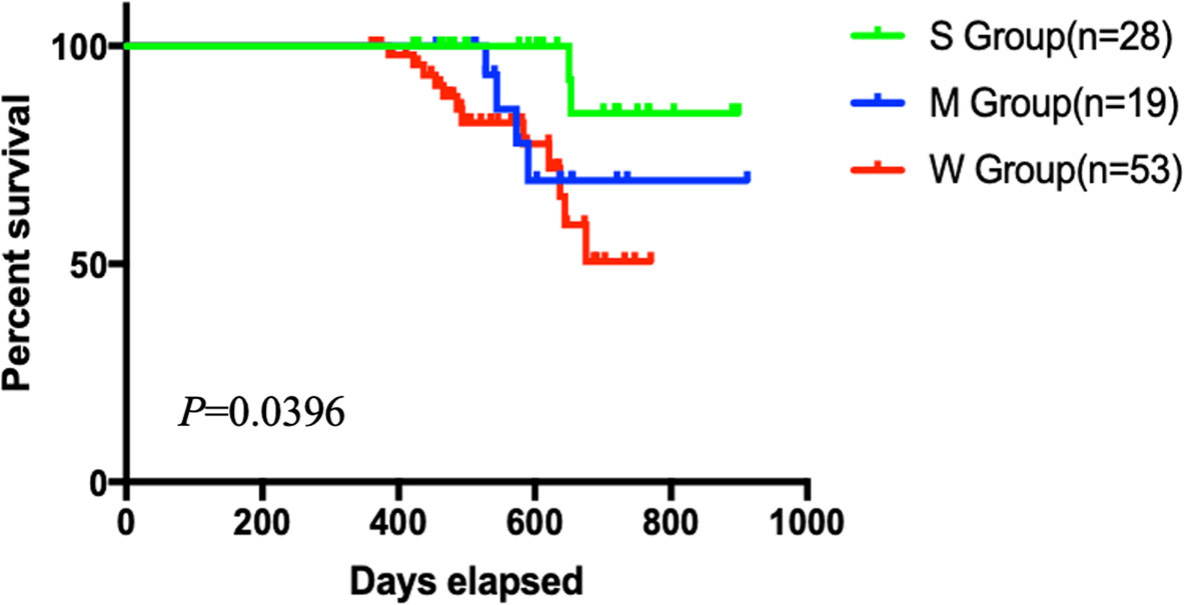
WNT7b activation was reversely associated with prognosis of the CRC cohort. Post-surgical survival rates analysis exhibited significantly differentiated prognosis between subgroups. Log-rank test, *P* = 0.0396

To examine the prognostic values of the multiple WNT7b upregulation levels identified above, we used Kaplan–Meier survival analysis. As shown in Fig. 6, post-surgical survival rates analysis showed significantly differentiated outcomes among subgroups. High levels of WNT7b upregulation predicted worst post-surgical survival rates. This finding supported our hypothesis that activation of WNT7b autocrine was an intrinsic step in the procedure of CRC progression and predicted poor outcome in CRC patients.

## Discussion

CRC is driven by certain oncogenes and genetic changes. Recent studies reported large-scale screening focused on WNTs and the Wnt/β-catenin cascade in CRC. Ruan et al. reported that the WNTs family mRNA expression was related to the diagnosis and prognosis of CRC. They analyzed RNA sequencing data from The Cancer Genome Atlas (TCGA) and showed that WNT2 and WNT7b had high diagnostic values in CRC. Their comprehensive prognosis analysis suggested that expression of WNT10B might serve as an independent prognostic biomarker of CRC [16]. Kleeman et al. analyzed 1262 colorectal cancer cases and found that epigenetic suppression of appropriate WNT negative feedback loops were selectively advantageous in ligand-dependent (LD) tumors (identifiable mutations in APC or CTNNB1). They suggest that distinguishing between LD and ligand-independent (LI) tumor types is important; patients with LD tumors retained sensitivity to WNT ligand inhibition and may be stratified at diagnosis to clinical trials of porcupine inhibitors [17]. As more attention turned to WNTs, there were studies of WNT isoform mechanisms in CRC. Aizawa et al. isolated cancer-associated fibroblasts (CAFs) and normal fibroblasts (NFs) from surgical resected CRC tissues and found that WNT2 protein released from CAFs enhanced CRC cell invasion and migration, playing a key role in cancer progression emerging as a potential therapeutic target for CRC [18]. Galbraith et al. and Peng et al. both argued that WNT6a was associated with liver metastasis of CRC and predicted poor outcomes in CRC patients [19, 20]. In the present study, we were the first to profile WNT7b expression patterns and levels in CRC. We demonstrated that the activation of WNT7b secretion leads to metastasis and predict poor outcome. In mechanistic studies, we found that high levels of WNT7b secretion were associated with activation of EMT through the canonical Wnt/β-catenin signaling pathway.

The β-catenin dependent signaling pathway is triggered by the binding of Wnt ligands to the LRP-5/6 receptors (low-density lipoprotein receptor) and Frizzled receptors. This in turn activates Disheveled (DVL), causing recruitment of the complex (Axin, GSK3β, CK1, APC) to the receptor [21–23]. The Wnt-Frizzled-Axin -LRP-5/6 complex sequesters cytosolic GSK3β, rendering it incapable of phosphorylating β-catenin. The accumulation of unphosphorylated β-catenin in the cytosol migrates to the nucleus, interacting with T cell-specific factor (TCF)/lymphoid enhancer-binding factor (LEF) and co-activators to turn on the Wnt target genes such as c-Myc, cyclin D1 and Cdkn1a, thereby enabling tumor cells to gain growth and metastatic dynamics.

Given the key role of the Wnt/β-catenin signaling pathway in cancer development and invasion, Wnt/β-catenin signaling pathway inhibitors were identified. Selective porcupine (PORCN) inhibitors, LGK974 and ETC-159 target the secretion of Wnt ligands; these are being studied in ongoing phase 1/2 trials in metastatic colorectal and head and neck cancers [24]. Specific Wnt ligands and receptors are found to be overexpressed in many tumors; monoclonal antibodies developed against Wnt-1 and Wnt-2 demonstrate Wnt inhibition leading to tumor suppression in melanoma, sarcoma, colorectal cancers, non-small cell lung carcinoma, and mesothelioma [25, 26]. However, there are no drugs approved to target Wnt/β-catenin, although it has been a compelling target for inhibition in past 30 years. The present study provides an alternative choice for precision therapy of CRC or for combined therapeutic approaches. More evidence is needed.

A major unresolved question in this study is how the expression of WNT7b ligand is controlled, and the upstream signaling regulation remains unclear. Indeed, data regarding upstream regulation of the Wnt signaling pathway is limited. A recent study from Moparthi et al. found that FOXB2, an uncharacterized protein, is a potent regulator of Wnt ligand expression and TCF signaling that drives the neuroendocrine differentiation of prostate cancer cells [13]. Another study also reported that MM-1 competitively binds the *wnt4* gene promotor region, thereby downregulating promoter activity of *wnt4* gene expression [27]. However, WNT7b and its co-receptors are characterized by tissue specific restriction. In particular, WNT7b elicits limited pathway activation on its own, as evidenced by its inability to induce LRP6 phosphorylation and β-catenin stabilization to any substantial degree [28]. By contrast, WNT7b strongly cooperates with other ligands, primarily WNT1, in driving TCF/LEF-dependent gene transcription. The mechanism of the WNT7b-dependent pathway activation is unclear; however, it requires additional coreceptors, namely RECK and GPR124 [29, 30]. Our understanding of WNT7b signaling in CRC is evolving as all the samples from the CRC cohort presented strong WNT7b enrichment in the CRC tissues. Moreover, clinical data revealed that high levels of WNT7b activation contributed to the risk of metastasis. Future studies will focus on elucidating mechanisms that regulate higher levels of autocrine WNT7b signaling in CRC.

## Conclusion

The activation of WNT7b autocrine probably contributes to CRC metastasis by activating EMT through the Wnt/β-catenin signaling pathway. High levels of the WNT7b autocrine secretion predicts poor outcome in patients with CRC. WNT7b could be a promising candidate molecule in clinical CRC treatments.

## Supplementary Information

**Additional file 1: Supplementary Data 1.** Antibody list.

**Additional file 2: Supplementary Data 2.** WNT7b RNA interference sequence and primer sequence.

**Additional file 3: Supplementary Data 3.** Validation of the specificity of WNT7b siRNA by qRT-PCR analysis.

**Additional file 4: Supplementary Figure 1.** Original images for blot and gel figures.

## Data Availability

The datasets during the current study are available from the corresponding author on reasonable request.

## Abbreviations

CRC: Colorectal cancer
EMT: Epithelial–mesenchymal transition
IHC: Immunohistochemistry staining
H&E: Hematoxylin-eosin
TMAs: Tissue microarrays
AJCC: American Joint Committee on Cancer
DMEM: Dulbecco’s Modified Eagle’s Medium
DKK1: Dickkopf-1
TCGA: The Cancer Genome Atlas
LD: Ligand-dependent
LI: Ligand-independent
CAFs: Cancer-associated fibroblasts
NFs: Normal fibroblasts
DVL: Disheveled
TCF: T cell-specific factor
LEF: Lymphoid enhancer-binding factor
