# Supplementary material for: Activation of WNT7b autocrine eases metastasis of colorectal cancer via epithelial to mesenchymal transition and predicts poor prognosis
Source: BMC Cancer. 2021 Feb 19;21:180. doi: 10.1186/s12885-021-07898-2 (PMC7893751; doi:10.1186/s12885-021-07898-2)
Supplement: Supplementary file 3 — Additional file 3: Supplementary Data 3. Validation of the specificity of WNT7b siRNA by qRT-PCR analysis. [file 12885_2021_7898_MOESM3_ESM.pdf]

Supplemental Data 4

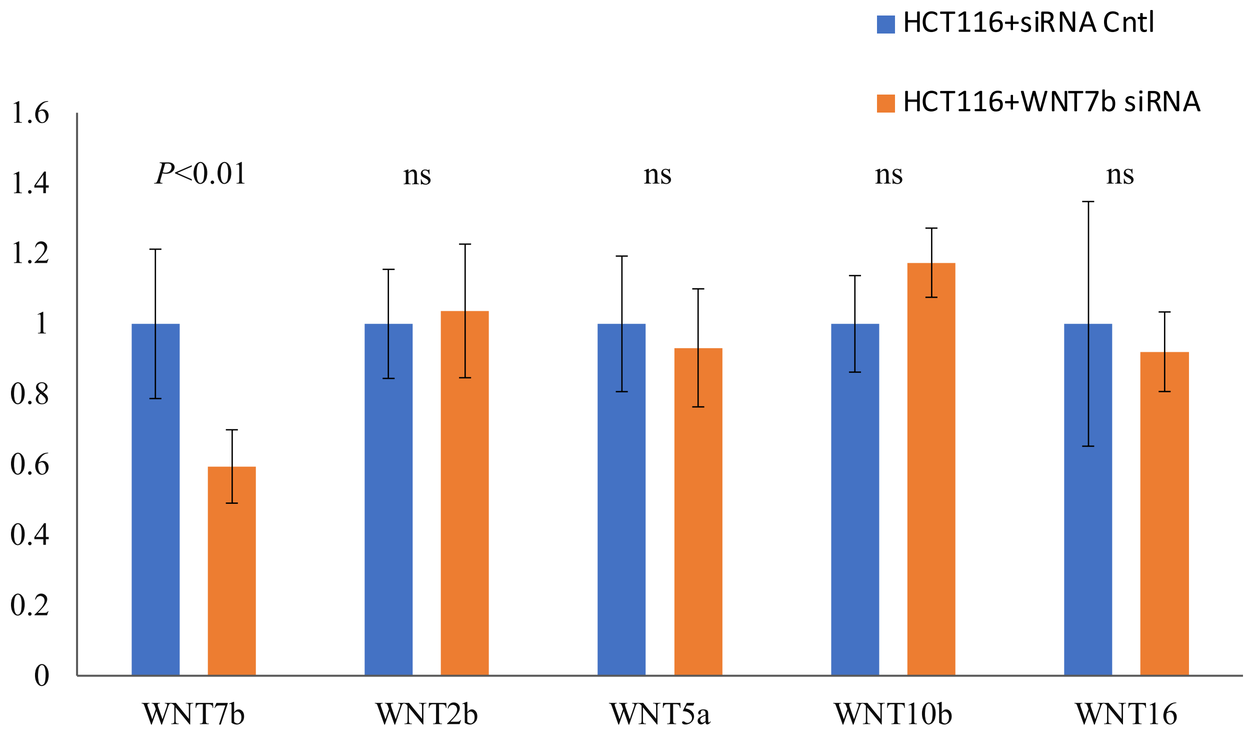

Supplemental Data 4. Specificity of WNT7b siRNA was checked by qRT-PCR. WNT7b, WNT2b, WNT5a, WNT10b, and WNT16 transcriptional level in HCT116 NC group and HCT116 WNT7b knock-down group were checked by qRT-PCR analysis. P was calculated by two tails student's t test.
