# Supplementary material for: Activation of WNT7b autocrine eases metastasis of colorectal cancer via epithelial to mesenchymal transition and predicts poor prognosis
Source: BMC Cancer. 2021 Feb 19;21:180. doi: 10.1186/s12885-021-07898-2 (PMC7893751; doi:10.1186/s12885-021-07898-2)
Supplement: Supplementary file 4 — Additional file 4: Supplementary Figure 1. Original images for blot and gel figures. [file 12885_2021_7898_MOESM4_ESM.pdf]

Activation of WNT7b Autocrine Eases Metastasis of Colorectal Cancer via Epithelial to Mesenchymal Transition and Predicts Poor Prognosis

Additional data

Blot images interpretation

Shuai Jiang; Qiwen Li; Yimin Liu; Huimin Zhang; Qianyu Wang; Yu Chen; Xiaoyang Shi; Jun Li; Hailing Zhang; Yi Zhang; Dongqing Xia; Man Wu; Jiajia Lin; Chenglin Zhang; Suhua Pang; Jiamin Jiang; YAN WEN, M.D.; Peipei Zhang

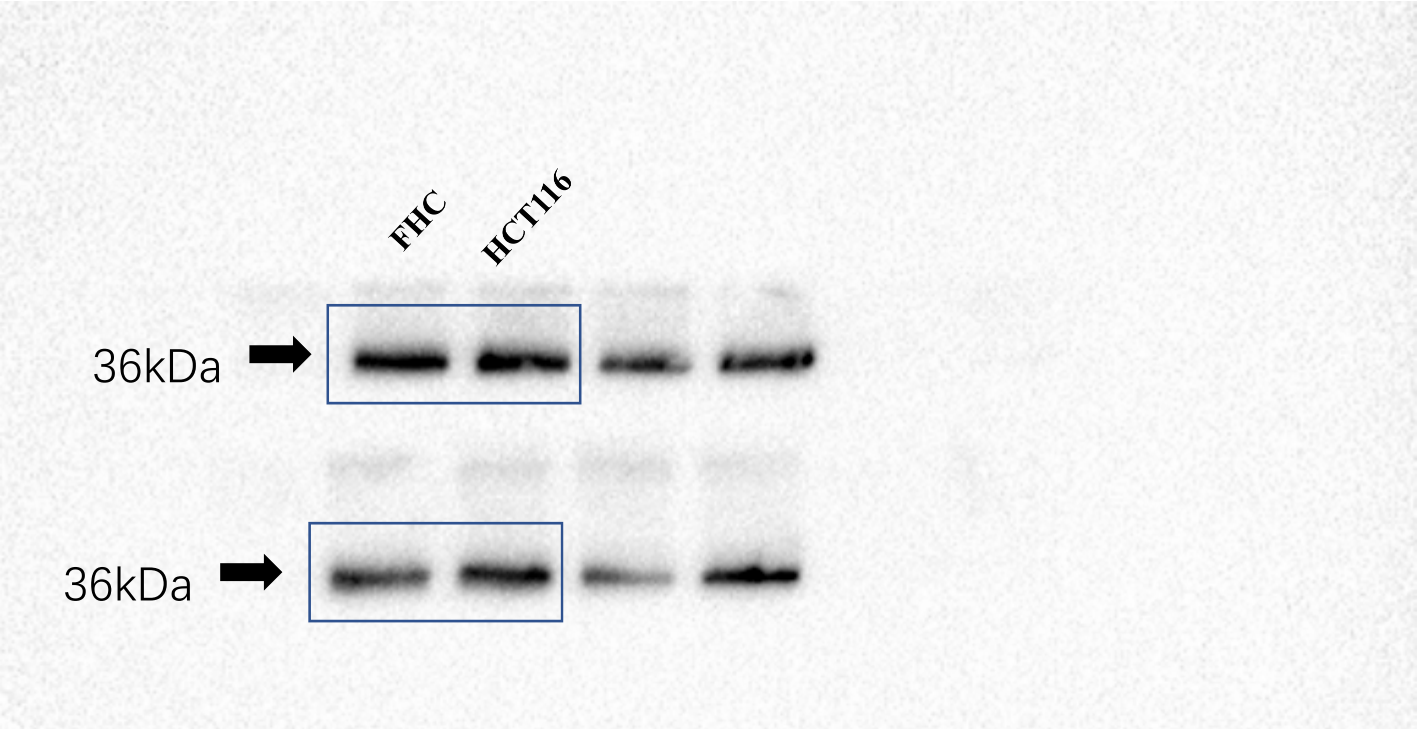

Fig. 3B and Fig.4A GAPDH

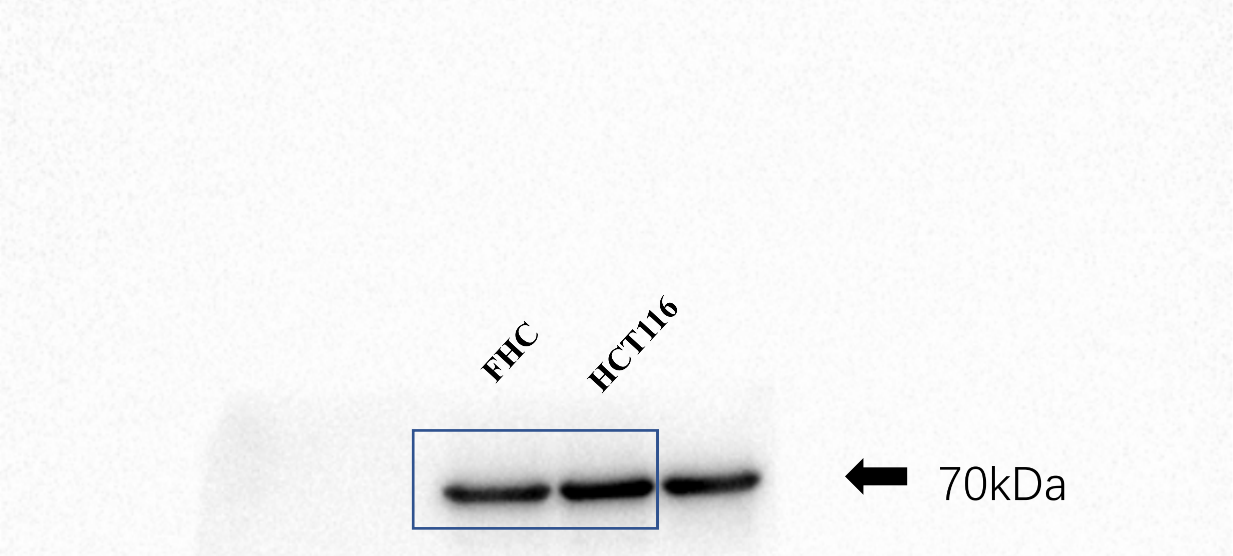

Fig. 3B Lamin B1

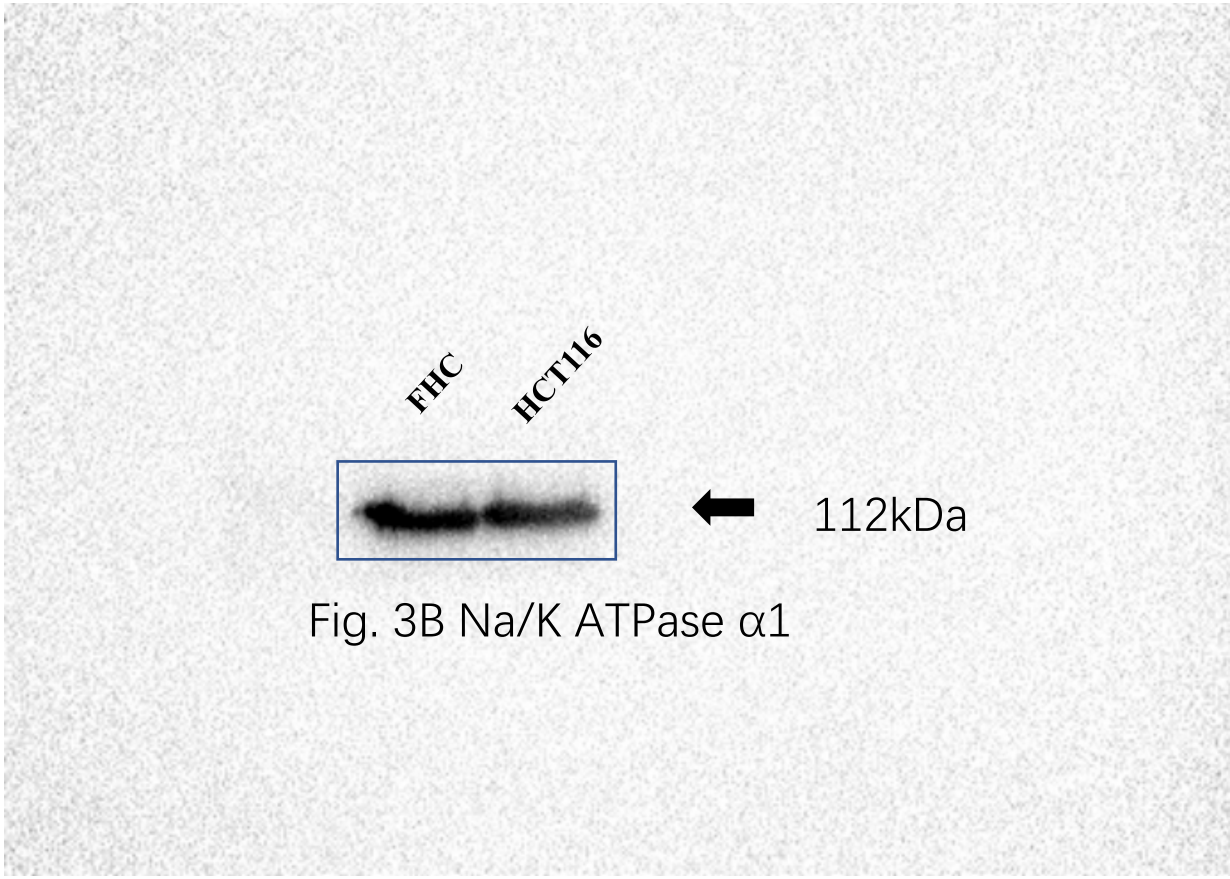

Fig. 3B Na/K ATPase α1

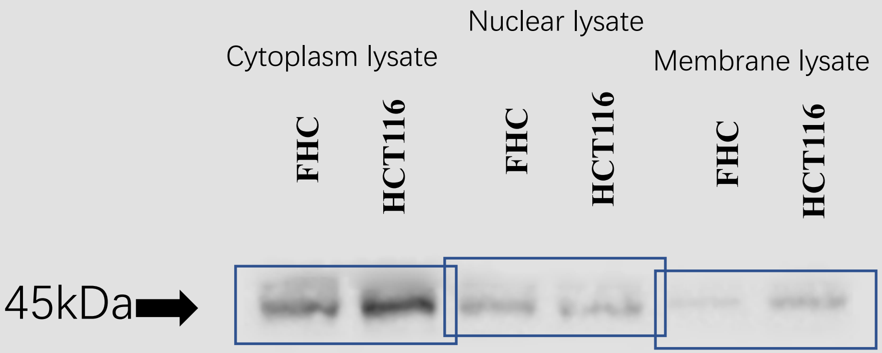

Fig. 3B cytoplasm/nuclear lysate/membrane WNT7b

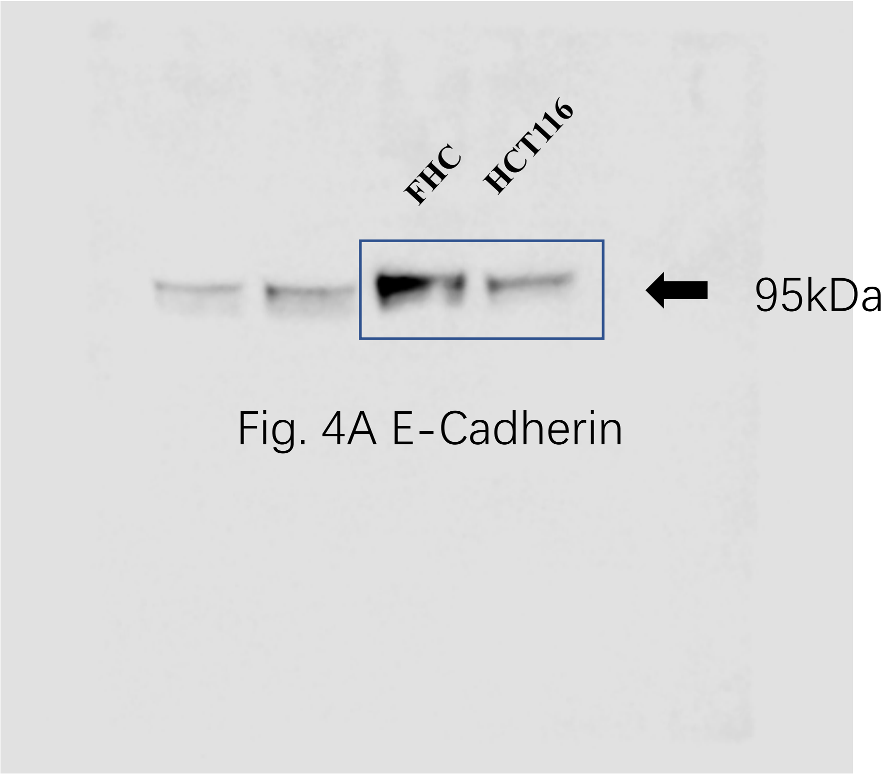

Fig. 4A E-Cadherin

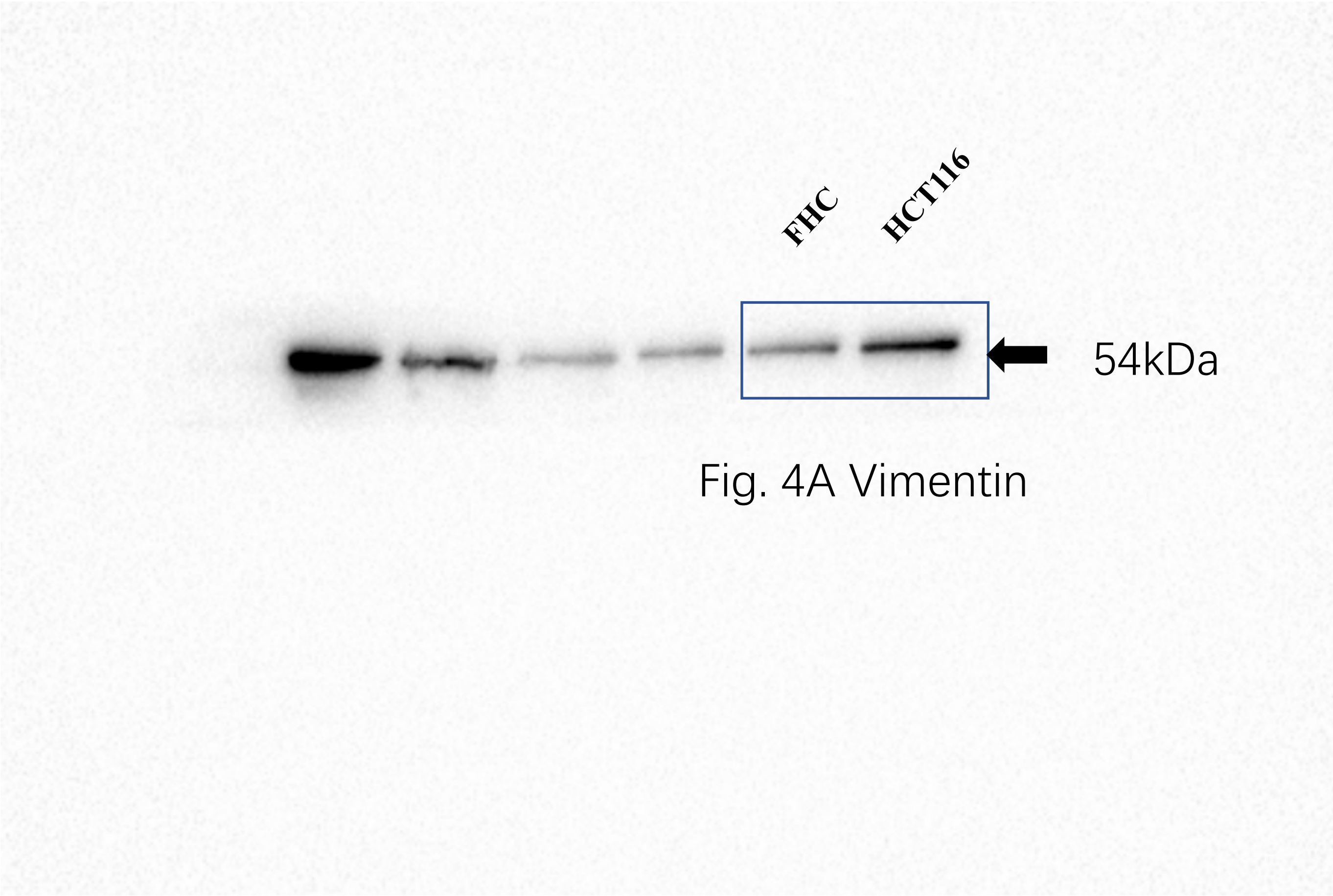

Fig. 4A Vimentin

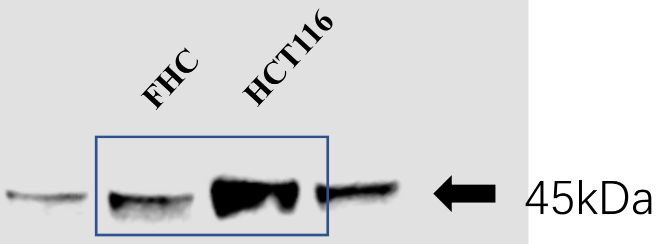

Fig. 4A WNT7b

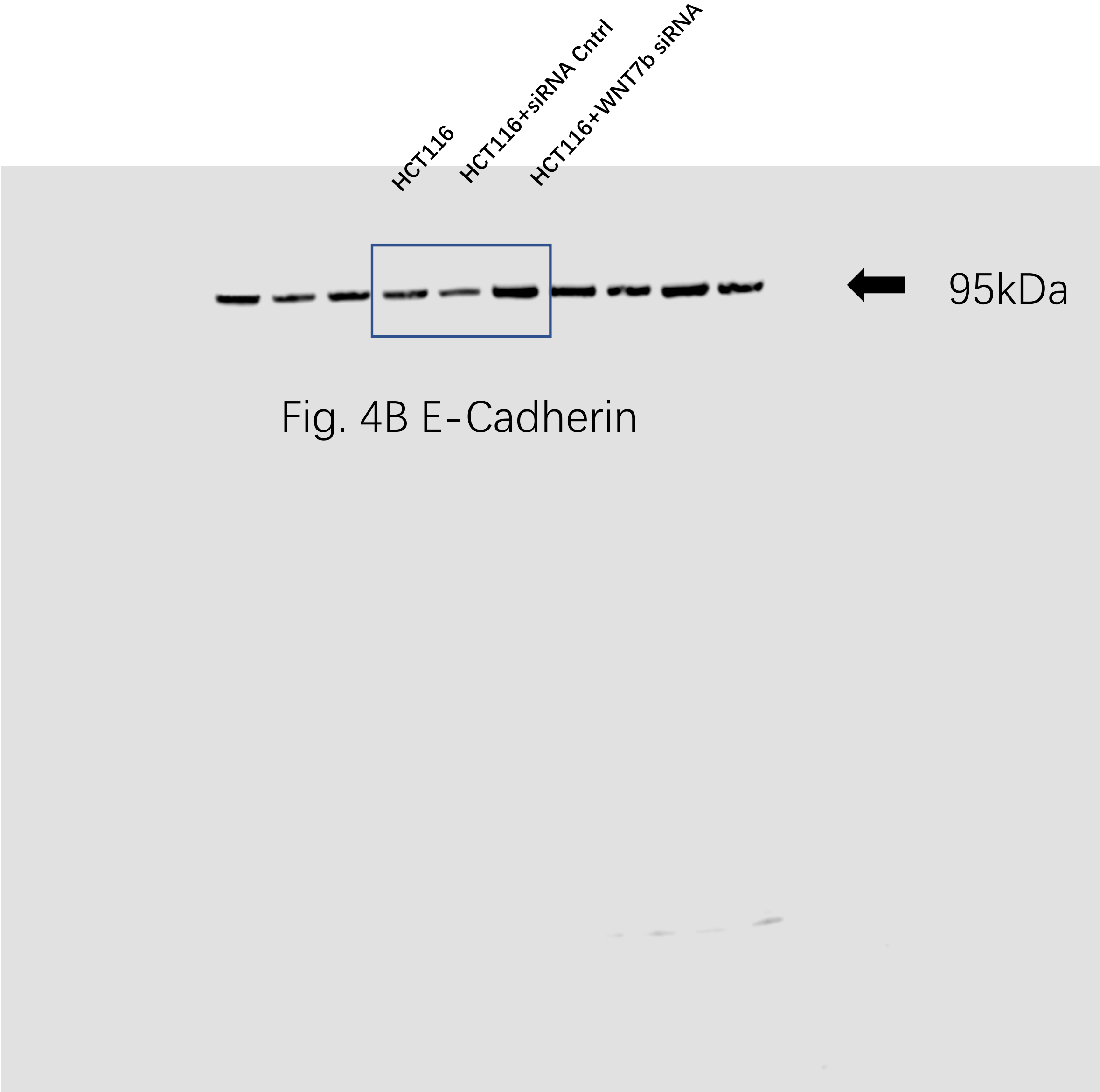

Fig. 4B E-Cadherin

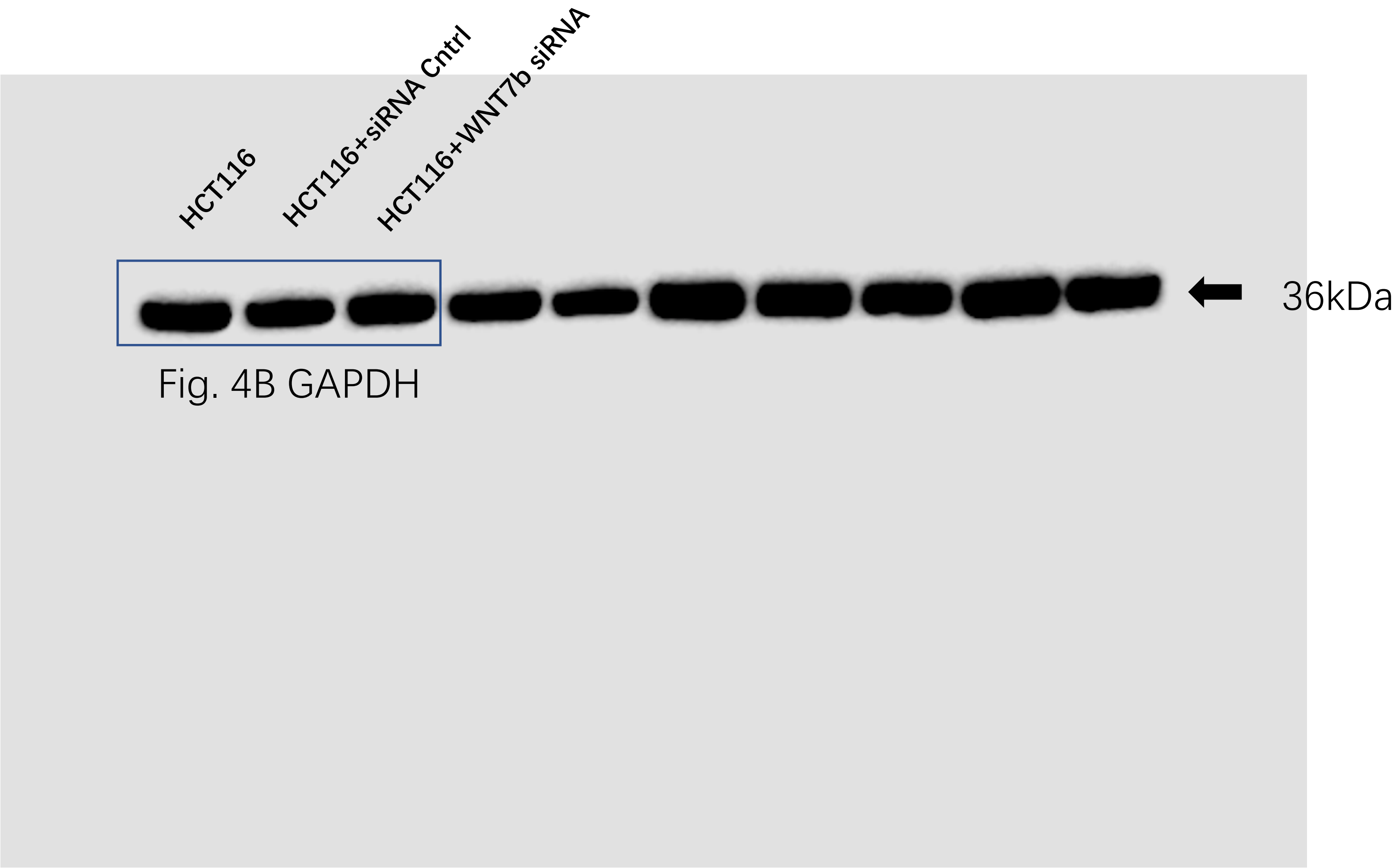

Fig. 4B GAPDH

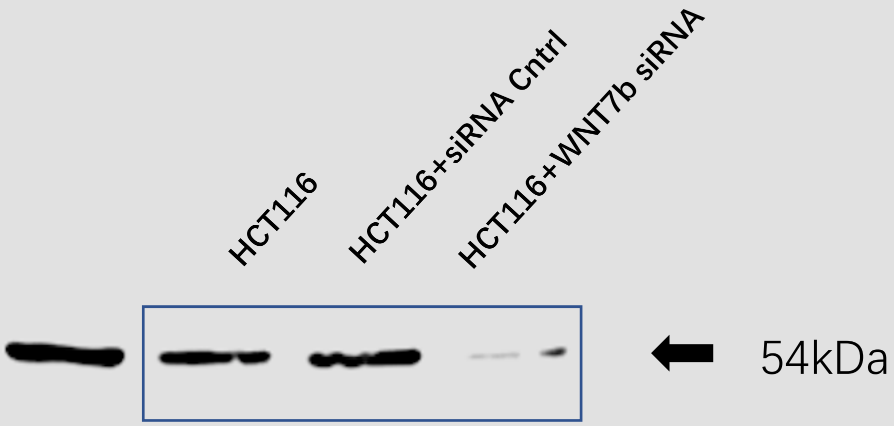

Fig. 4B Vimentin

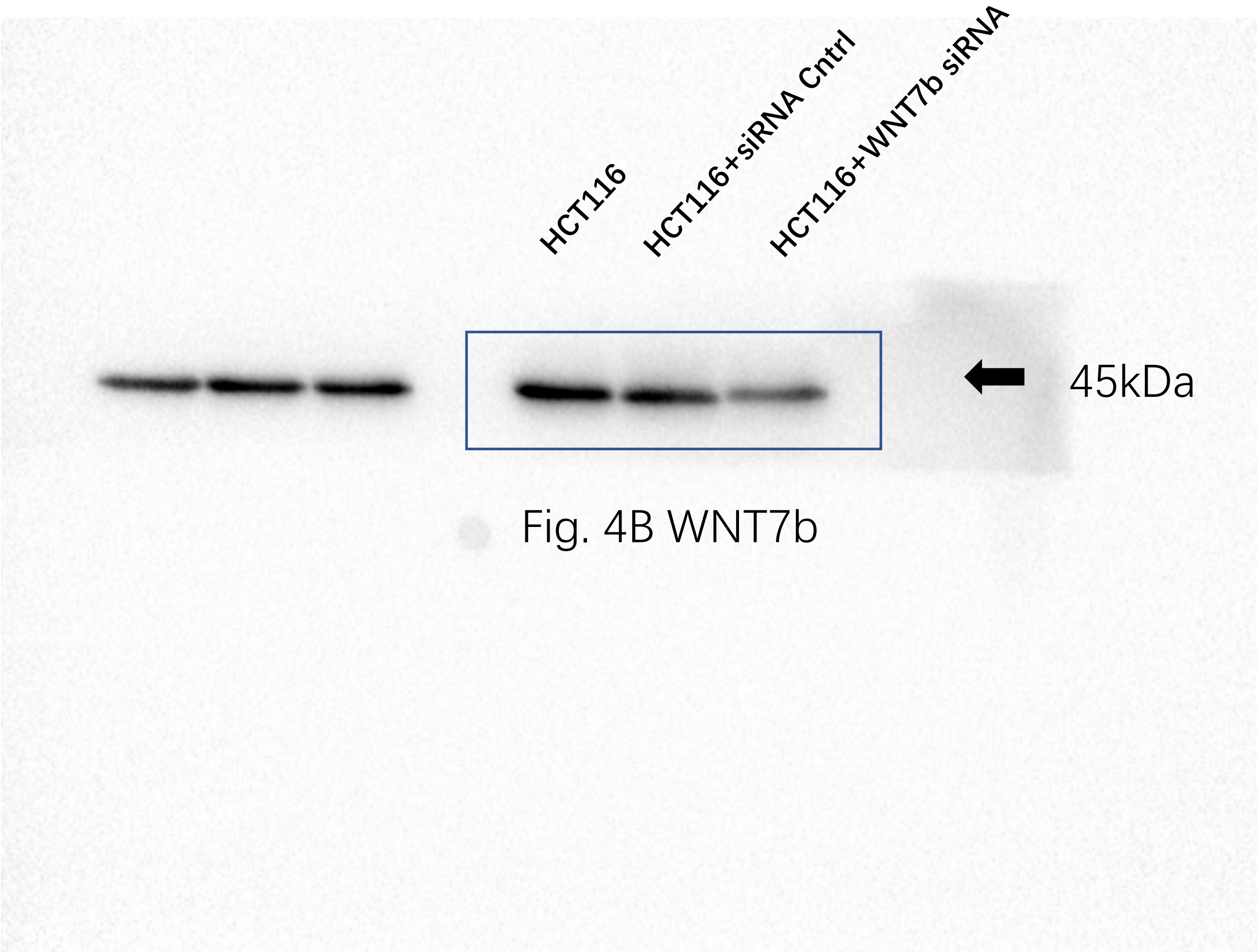

Fig. 4B WNT7b

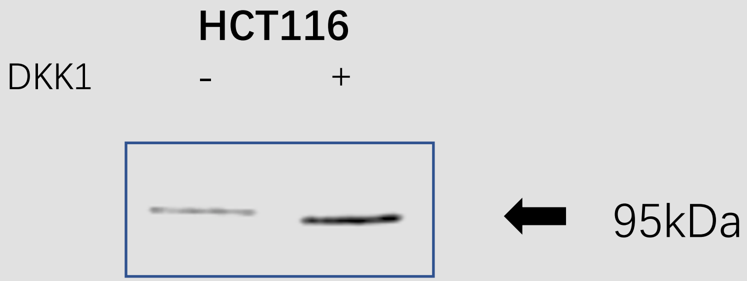

Fig. 4C E-Cadherin

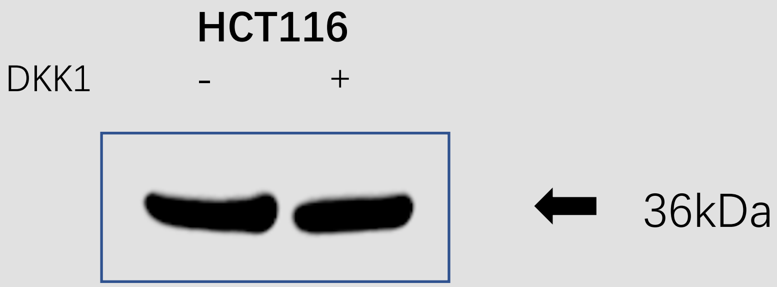

Fig. 4C GAPDH

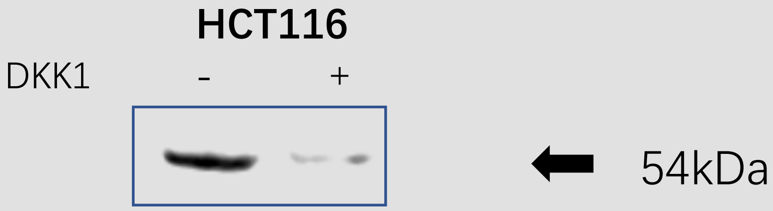

Fig. 4C Vimentin

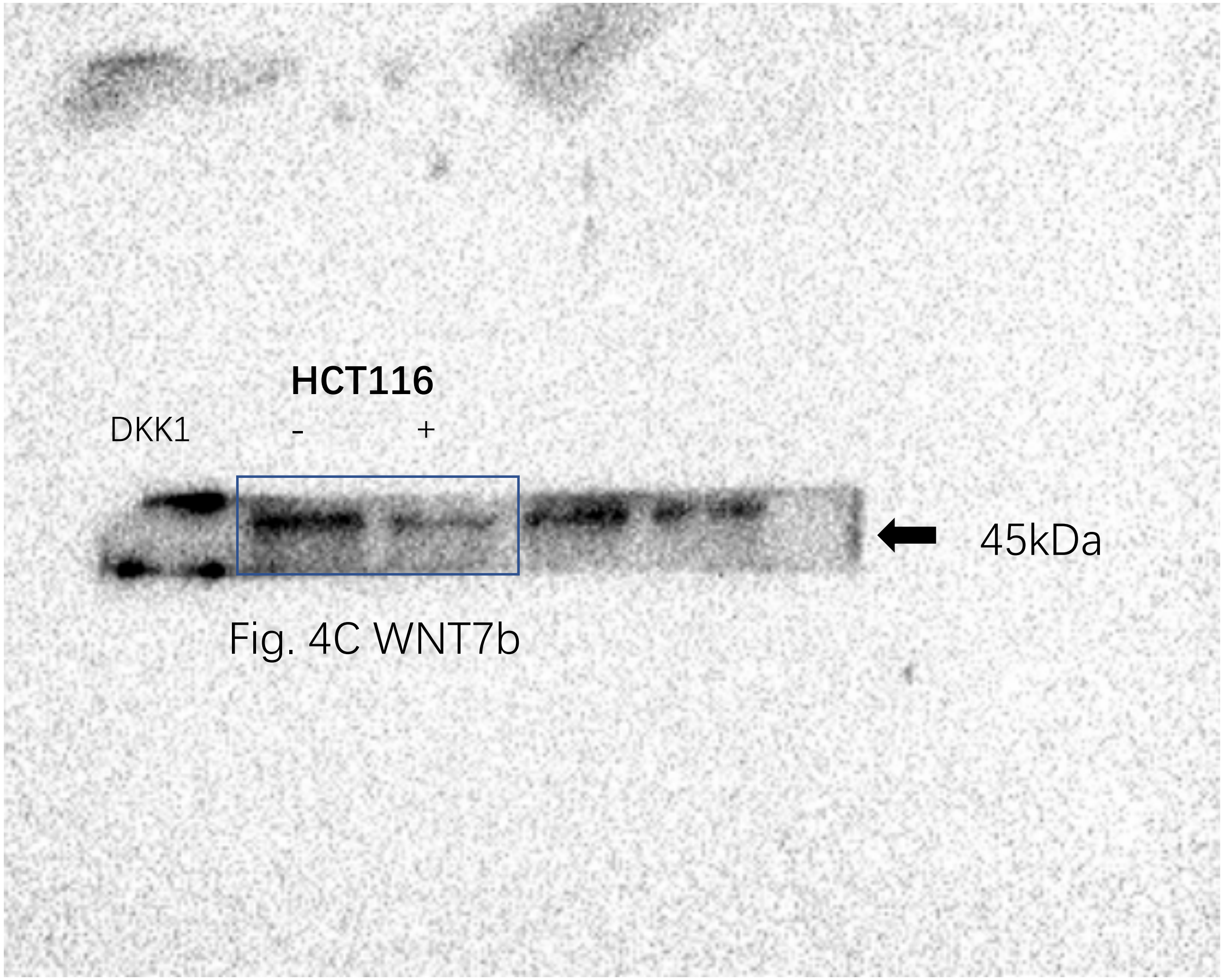

Fig. 4C WNT7b
